# Avoiding erroneous decisions: evidence, population impact and acceptability of universal mental health interventions in schools – a commentary on Andrews et al.

**DOI:** 10.1111/camh.70126

**Published:** 2026-09-20

**Authors:** Chris Bonell, Kelly Rose‐Clarke, Jess Deighton, Graham Moore, Willem Kuyken, Mark Greenberg

**Affiliations:** ^1^ Public Health & Sociology, Department of Public Health, Environments & Society London School of Hygiene & Tropical Medicine London UK; ^2^ Global Mental Health, Institute for Global Health University College London London UK; ^3^ Child Mental Health & Wellbeing University College London London UK; ^4^ DECIPHer, School of Social Sciences Cardiff University Cardiff UK; ^5^ Department of Psychiatry University of Oxford Oxford UK; ^6^ Edna Bennett Pierce Prevention Research Center, College of Health & Human Development Pennsylvania State University University Park Pennsylvania USA

**Keywords:** Universal, school, prevalence inflation, harm, impact

## Abstract

Foulkes and colleagues argue many ‘universal’ interventions to prevent mental health problems are harmful (‘inflating prevalence’ because they raise awareness of mental health problems, encouraging those without such problems to misinterpret everyday concerns as symptoms of mental health disorders, causing avoidance of stressful situations, worsening mental health), relatively ineffective (effect sizes being smaller than for targeted interventions) and/or unacceptable to students, and should be discontinued. These arguments are based on weak evidence. While their ‘prevalence inflation’ hypothesis is potentially important and plausible when applied to awareness‐raising interventions, evidence for it is currently weak and there is a tendency to overgeneralise to broader interventions. Arguments about the relative impacts of universal and targeted interventions fail to distinguish between individual and population impacts. Arguments about unacceptability are based on selected findings from a few studies, not reflecting broader evidence. These arguments could lead to erroneous decisions to discontinue universal mental health interventions in schools when they are most needed.

## Introduction

Schools are increasingly sites for ‘universal’ interventions aiming to prevent mental health problems and promote well‐being among all students. Foulkes and colleagues argue that many such interventions are harmful, ineffective and/or unacceptable to students, and should be discontinued (Andrews & Foulkes, 2025; Foulkes & Andrews, 2023). Their first argument, worded in terms of a ‘prevalence inflation hypothesis’, is that interventions specifically aiming to raise student awareness of mental health problems inadvertently encourage those initially without such problems to misinterpret everyday concerns as symptoms of mental health disorders, leading to avoidance of stressful situations, worsening mental health (Foulkes & Andrews, 2023). Their second argument is that universal interventions are minimally impactful compared with targeted support for those with mental health problems (Andrews & Foulkes, 2025). Their third argument is that such interventions are unacceptable to students (Andrews & Foulkes, 2025). Foulkes and colleagues recommend a shift from universal interventions towards those addressing factors such as bullying that increase risk of mental health problems, supporting children and young people at increased risk of mental health problems, and referring those with mental health disorders for treatment outside school (Andrews & Foulkes, 2025). These arguments have evoked considerable media attention and interest from policymakers.

We contend these arguments are based on weak evidence. While we agree the prevalence inflation hypothesis is potentially important and plausible when applied to awareness‐raising interventions, we argue evidence for it is currently weak and there is a tendency for consideration to overgeneralise from awareness‐raising interventions to a broader set of interventions. We suggest arguments about the relative impacts of universal and targeted interventions fail to distinguish between individual and population impacts. Third, we highlight that arguments about unacceptability are based on a few studies, selectively reported and do not reflect broader evidence. We fear the arguments, as currently worded, could lead to erroneous decisions to discontinue universal mental health interventions in schools when they are most needed.

## Evidence awareness‐raising interventions cause harm is weak

Foulkes and team offer three pieces of evidence to support the prevalence inflation hypothesis, but all have important limitations.

First, they cite (Andrews & Foulkes, 2025) an existing systematic review of school cognitive behavioural therapy (CBT) by Guzman‐Holst et al., interpreting this as indicating CBT in schools causes increases in depression and anxiety. However, this systematic review focused only on school bullying prevention interventions, including targeted and universal interventions. Two of seven included studies using CBT elements reported negative effects, while others reported positive impacts. The former two were targeted, not universal interventions. The review did not focus on school CBT to promote mental health and did not include all studies of this. Systematic reviews of such studies generally report positive effects (Tse et al., 2023).

Second, Foulkes and team cite (Andrews & Foulkes, 2025) the My Resilience in Adolescence (MYRIAD) trial reported by Kuyken et al., which assessed the impact of a mindfulness intervention for students in English secondary schools. The trial included planned analyses examining intervention effects on 3 primary and 28 secondary outcome measures at two follow‐up points, with the one‐year follow‐up being primary. For five of the 62 significance tests, there was evidence of intervention students doing slightly worse on some secondary outcomes. Exploratory subgroup analyses found intervention students with higher baseline risk of mental health problems did slightly worse than controls on the primary depression and well‐being outcomes. The effects reported in the planned analyses are all extremely small with no consistent pattern. The subgroup effects are somewhat larger with a more consistent pattern. Overall, the study offers no evidence that the intervention was harmful overall on the primary outcomes and some signal that the intervention group did somewhat worse where they reported elevated baseline risk. Few students exposed to the intervention reported practising mindfulness outside classrooms, with particularly low adherence among students with elevated baseline risk as reported by Montero‐Marin et al. Nine significant results arose from 104 tests: only slightly more than would be expected from chance. Forthcoming results from the planned 2‐year follow‐up suggest no overall positive or negative intervention effects (Kuyken, personal communication).

Third, Foulkes and colleagues cite a scoping review aiming to identify harmful school mental health interventions (Guzman‐Holst, Streckfuss Davis, Andrews & Foulkes, 2025). This found that 8.93% of 120 studies of such interventions reported evidence of harm for at least one outcome: six were of CBT and four of mindfulness (Guzman‐Holst et al., 2025). The rate and pattern of reported harms align with an expectation of one chance false‐positive result per 20 tests. Furthermore, some studies classified as reporting harms also reported benefits. Klim‐Conforti et al.'s trial of universal CBT found that while male (not female) students reported higher impulsivity, there were reduced overall rates of suicidal ideation (primary outcome) and improvements in depression, anxiety, emotional regulation, self‐concept and inter‐personal difficulties. Seely et al.'s trial of CBT reported less prosocial behaviour but also less hyperactivity in the intervention group. Frank et al.'s trial of universal teacher‐delivered mindfulness reported less goal‐directed behaviour but more executive functioning and working memory in the intervention arm. A better approach would have been to employ standard systematic methods to pool overall evidence of the effects of CBT and mindfulness on anxiety and depression to assess the balance of harm, benefit or null effect.

As well as each being weak in demonstrating harms, the above evidence does not align with the prevalence inflation hypothesis. The hypothesis focuses on *awareness‐raising* interventions for those *without prior mental health problems*. However, neither CBT nor mindfulness aims to raise awareness of mental health problems, and much of the evidence of harm is from subgroups with *worse* prior mental health.

Nonetheless, we think the prevalence inflation hypothesis is plausible when applied to awareness‐raising interventions. Two recent English trials offer some support (Deighton et al., 2025). The Youth Aware of Mental Health intervention aimed to improve understanding of mental health. It was found to increase young people's risk of emotional difficulties at 1‐year follow‐up. The Mental Health and High School Curriculum Guide intervention involved lessons explaining common mental disorders and was found to increase the risk of emotional difficulties and decreased life satisfaction at 1‐year follow‐up. This evidence potentially supports the prevalence inflation hypothesis because the focus is on awareness‐raising interventions and evidence of harms among all students. Overall, while the prevalence inflation hypothesis is plausible in relation to the subset of universal awareness‐raising interventions, it needs to be rigorously tested through further trials of such interventions and should not be generalised to other universal school interventions that target entirely different mechanisms.

## Evidence for population impacts of universal interventions is insufficiently considered

Foulkes and colleagues' second argument, that universal school mental health interventions are minimally impactful, is based on evidence of smaller impact per student of universal CBT compared with targeted CBT in schools. They quote a review of universal CBT interventions reporting long‐term (>12 months) effect sizes of Hedges' *g* = 0.07 for depression and *g* = 0.09 for anxiety (Andrews & Foulkes, 2025; Werner‐Seidler et al., 2021). Our reading of this review is that the figures reported are *g* = 0.10 and *g* = 0.11, respectively (Werner‐Seidler et al., 2021). The review reported the effects of: *g* = 0.21 for depression and *g* = 0.18 for anxiety immediately post‐intervention, *g* = 0.17 and *g* = 0.19 for 0–6‐month follow‐up, and 9 = 0.10 and *g* = 0.23 for >6–12‐month follow‐up for these outcomes (Werner‐Seidler et al., 2021).

It is unsurprising that targeted interventions have bigger impacts than universal interventions per individual because there is more room for improvement among those with existing problems. However, it is also well established that population impacts of universal interventions can be disproportionate to benefits per individual. The Rose hypothesis suggests that, where risks are normally distributed in a population, universal interventions generating less benefit per individual still avert more adverse outcomes per population than do targeted interventions because most adverse outcomes arise from individuals at low or medium risk (there being more of these) which universal interventions reach but targeted interventions do not. In the case of universal school CBT, a population‐wide shift of 0.10 standard deviations would meaningfully reduce the proportion of individuals at highest risk. Thus, the effects reported for CBT impacts on anxiety and depression are likely consistent with important population impacts.

Furthermore, Foulkes and colleagues focus on CBT and mindfulness interventions but not other interventions, such as social and emotional learning (SEL). There have been >10 meta‐analyses of >500 universal SEL interventions reporting positive effects on mental health, social competence and well‐being. The most recent meta‐analysis reported benefits related to child well‐being, competence, mental health and academic success (Cipriano et al., 2023). Broader reviews of universal interventions to prevent anxiety and depression suggest small but significant effects (Clarke et al., 2021; Hayes et al., 2025). Thus, to conclude universal interventions are insufficiently effective is a broad generalisation not supported by the evidence. There is also a risk that Foulkes et al.'s second argument implies an either/or choice between universal and targeted interventions when both likely have a role.

## Little evidence that universal prevention is unacceptable to young people

Foulkes and colleagues' third argument is that young people do not enjoy or want universal mental health interventions in schools (Andrews & Foulkes, 2025). Evidence supporting this comes from just two papers, selectively reported. Foulkes and team report that only 49% of recipients of an Australian universal online intervention thought the intervention completely or somewhat relevant to their lives, and 29% would recommend it to their friends, but not that 60% rated it as good or very good, and 69% felt it would help them somewhat or a great deal to deal effectively with future problems. Foulkes and colleagues report evidence from a qualitative analysis (focused exclusively on negative student assessments) from the MYRIAD trial but not that other MYRIAD analyses identify a balance of positive and negative student assessments. Thus, these two studies do not support Foulkes and colleagues' argument. Broader qualitative evidence suggests that universal school interventions are acceptable to students (National Institute for Health and Care Excellence, 2022).

## Conclusion

First, there is a risk that the suggestion of harms encourages policymakers and schools to disinvest from promoting young people's mental health. This is contrary to multiple studies of various universal interventions which report no evidence of harm and important benefits for reducing problems such as anxiety and depression (Cipriano et al., 2023; Clarke et al., 2021; Hayes et al., 2025). Nonetheless, it is important to consider and further examine the potential for harm, especially for interventions focused on raising mental health awareness. But when overgeneralised to all universal mental health interventions in schools, the prevalence inflation theory likely represents an example of exaggerated unintended effects. Second, it is important to recognise the potentially transformatory population impacts of universal school mental health interventions even when benefits per individual may not be as great as for targeted interventions. Third, we cannot conclude that universal school mental health interventions are negatively rated by students based on just two studies, when these studies actually suggest a balance between positive and negative ratings and with broader evidence suggesting universal interventions are acceptable to students (National Institute for Health and Care Excellence, 2022).

With recent reports indicating a continuing increase in mental health problems among children and young people, it has never been more crucial that we ensure that the help and support on offer is timely, acceptable and effective. Arguments made within academia that cause us to question our approach can be helpful in encouraging scrutiny, ensuring interventions meet these needs. However, as detailed above, these recent challenges have significant limitations and risk undermining efforts to ensure evidence‐based and effective practice is promoted and implemented within schools. It is critically important that policymakers and schools do not discontinue universal mental health interventions based on weak evidence at the very time when there is an epidemic of mental ill‐health among children and young people. A longer version of this paper is available from the first author on request.

## Funding information

The authors declare this paper was written without funding.

## Conflict of interest statement

The authors declare no conflicts of interest.

## AI use statement

The authors declare that they have not used AI in any capacity that would require disclosure in accordance with Wiley's AI Guidelines, nor in any capacity that would reasonably require disclosure for editors, reviewers and readers to properly evaluate their research or manuscript. The authors confirm that AI has not been used for purposes including but NOT limited to drafting and editing, to generate substantial text or restructure arguments, nor in the research methodology. The authors take full responsibility for the accuracy of this statement.

## Ethics statement

Ethics approval is not applicable to this Commentary article.

## Data Availability

Data sharing not applicable to this article as no datasets were generated or analysed for this Commentary article.
